# Crystal structure of 1,4-bis­[5-(2-meth­oxy­phen­yl)-2*H*-tetra­zol-2-yl]butane

**DOI:** 10.1107/S2056989019014877

**Published:** 2019-11-08

**Authors:** Young Min Byun, Farwa Ume, Ji Yeon Ryu, Junseong Lee, Hyoung-Ryun Park

**Affiliations:** a Department of Chemistry, Chonnam National University, Gwangju, 61186, Republic of Korea

**Keywords:** crystal structure, tetra­zole, bis-tetra­zol­yl, meth­oxy­lphenyl tetra­zole

## Abstract

The title compound, C_20_H_22_N_8_O_2_, was synthesized by the coupling reaction of a sodium tetra­zolate salt and di­bromo­butane in a molar ratio of 2:1. The reaction can produce several possible regioisomers and the title compound was separated as the major product. The X-ray crystallographic study confirmed that the title compound crystallizes in the monoclinic *P*2_1_/*c* space group and possesses a bridging butyl­ene group that connects two identical phenyl tetra­zole moieties. The butyl­ene group is attached not to the first but the second nitro­gen atoms of both tetra­zole rings. The dihedral angles between the phenyl groups and the adjacent tetra­zolyl rings are 5.32 (6) and 15.37 (7)°. In the crystal, the mol­ecules form centrosymmetric dimers through C—H⋯O hydrogen bonds between a C—H group of the butyl­ene linker and the O atom of a meth­oxy group.

## Abstract 2

The diffraction data confirmed the title compound as the main isomer produced in a coupling reaction. The structure and Hirshfeld surface analysis of the formed di-tetra­zolyl chelate ligand are reported.

## Chemical context   

Tetra­zole ligands have four nitro­gen atoms in their five-membered rings and the lone pairs of these nitro­gen atoms are useful for coordination bonds with metal ions (Zhao *et al.*, 2008 ▸). Tetra­zole has a variety of binding modes with metal ions, which results in the unusual formation of high-dimensional metal–organic frameworks (MOFs) or coordination polymers (Karaghiosoff *et al.*, 2009 ▸; Liu *et al.*, 2013 ▸). Valuable mono-, bis- and polytetra­zole ligands for the formation of MOFs and coordination polymers have been also reported (Boland *et al.*, 2013 ▸; Fan *et al.*, 2016 ▸; Tăbăcaru *et al.*, 2018 ▸; Zhao *et al.*, 2016 ▸). As an extension of a project on the study of self-assembly behaviour in solution, we designed a di­tetra­zolyl chelate ligand possessing a butane bridge. It is worth noting that tetra­zole has two different resonance structures in which the hydrogen atoms are located at either the N1 or N2 positions. In many cases, this results in the formation of several products (Lee *et al.*, 2017 ▸). It is therefore essential to study the mol­ecular structure of synthesized tetra­zole complexes by X-ray crystallography.
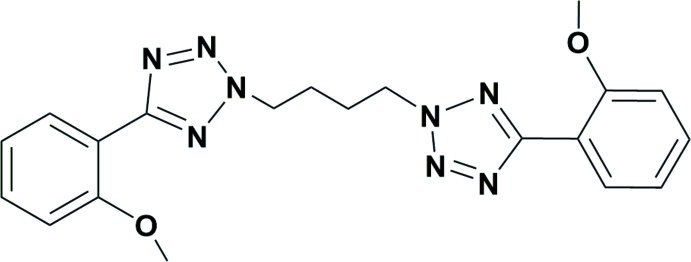


**Figure 1:**
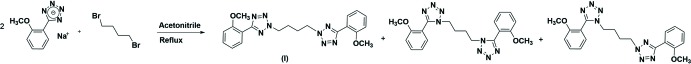
Synthesis of the title compound (I).

**Figure 2:**
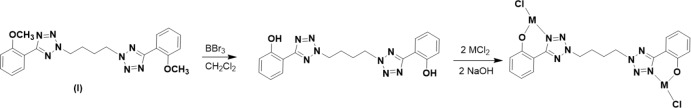
Synthetic route of the desired dinuclear metal complexes from the title compound (I).

The title compound was isolated as an inter­mediate in the middle of the synthetic route for a chelate ligand. The reaction between the sodium salt of tetra­zole and 1,4-di­bromo­butane gave three isomeric products (Fig. 1 ▸). Using column chromatography, the major product was isolated and its mol­ecular structure was determined unambiguously by X-ray crystallography. This compound is a useful precursor for the synthesis of dinuclear metal complexes with the expectation of synergetic effects of two metal centers (Fig. 2 ▸). Herein, we report the synthesis and crystal structure of this compound.

## Structural commentary   

**Figure 3:**
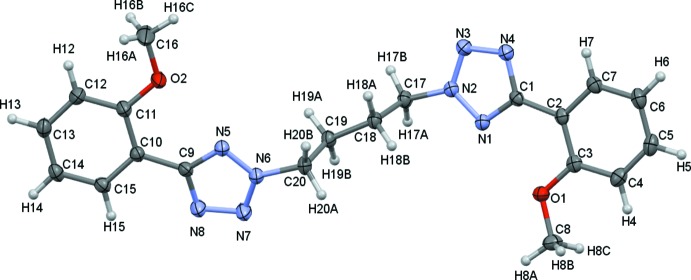
A view of the mol­ecular structure of the title compound, with the atom labelling and 30% probability displacement ellipsoids.

The reaction yielded three isomeric products as described in Section 5, *Synthesis and crystallization*, and the structural analysis confirms the formation of the desired major product. The mol­ecular structure of the title compound is shown in Fig. 3 ▸. There are no unusual bond lengths or angles. The title compound possesses two identical phenyl tetra­zole fragments, connected by a butyl (C17–C20) bridge. The butyl group is attached to the second N atom of both tetra­zole rings (N2 and N6, Fig. 3 ▸). The dihedral angles between the phenyl group and tetra­zolyl ring are somewhat different in the two phenyl­tetra­zolyl groups. One phenyl­tetra­zolyl group (N1–N4/C1–C7) is almost planar with an angle of 5.32 (6)° between the mean planes of the rings. However, the other phenyl­tetra­zolyl group (N5–N8/C9–C15) is tilted with a dihedral angle of 15.37 (7)°.

**Figure 4:**
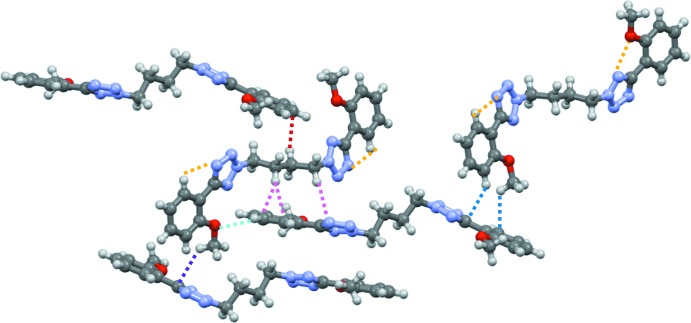
A plot showing the intra­molecular C—H⋯N hydrogen bonding (dashed yellow lines) and short contacts between mol­ecules (dashed pink, sky-blue and blue lines).

Two intra­molecular C—H⋯N hydrogen bonds (Table 1 ▸) occur, which are shown as yellow dashed lines in Fig. 4 ▸. These inter­actions may contribute to the planarity of the phenyl­tetra­zolyl units.

## Supra­molecular features   

**Figure 5:**
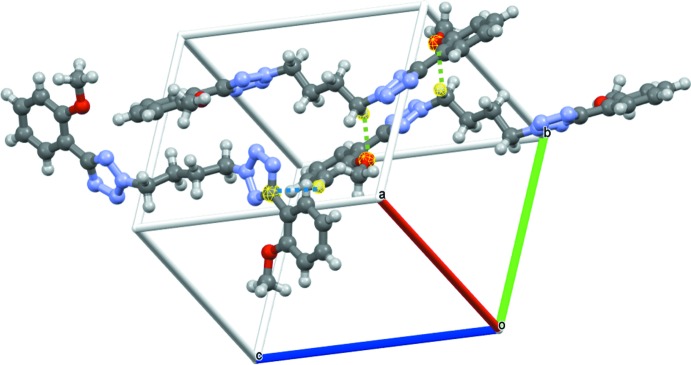
A plot showing the short contacts between mol­ecules (dashed green and blue lines).

The two phenyl­tetra­zolyl fragments exhibit different inter­molecular inter­actions. The tilted fragment (N5–N8/C9–C15) inter­acts with the butyl bridge of a glide-related mol­ecule through C19—H19*A*⋯C14^ii^ [H⋯*A* = 2.812 (2) Å; symmetry code: (ii) *x*, −*y* + 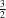, *z* + 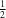], C19—H19*A*⋯C15^ii^ [H⋯*A* = 2.895 (2) Å] and C17—H17*B*⋯N8^ii^ [H⋯*A* = 2.729 (2) Å] contacts (Fig. 4 ▸, pink dashed lines). There is an additional weak C14^ii^—H14^ii^⋯O2 inter­action [H⋯*A* = 2.624 (2) Å] between the same pair of mol­ecules, which is indicated by a sky-blue dashed line in Fig. 4 ▸. The bridging butyl group forms a further C18—H18*B*⋯C5^iii^ [H⋯C = 2.738 (2) Å; symmetry code: (iii) *x*, −*y* + 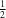, *z* − 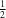] close contact (Fig. 4 ▸, red dashed line) with a mol­ecule generated by an adjacent glide plane. The planar fragments of screw-related mol­ecules form C4—H4⋯C1^iv^ [H⋯*A* = 2.692 (2) Å; symmetry code: (iv) −*x* + 2, *y* − 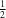, −*z* + 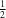] and C8—H8*C*⋯C7^iv^ [H⋯*A* = 2.828 (2) Å] close contacts, which are indicated by blue dashed lines in the right-hand side of Fig. 4 ▸ (for clarity a different reference mol­ecule was used for the illustration of this contact). It is inter­esting that the C1 atom has another close C—H⋯C contact from the opposite side of the aromatic plane (Fig. 4 ▸, purple dashed lines), C16—H16*A*⋯C1^v^ [H⋯C = 2.798 (2) Å; symmetry code: (v) −*x* + 1, *y* + 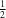, −*z* − 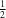]. There is one notable close contact, C17—H17*A*⋯O1^i^ that can be considered a weak hydrogen bond, which is indicated by green dashed line in Fig. 5 ▸. This contact forms a dimeric rectangle between two mol­ecules. This rectangle extends in the *c*-axis direction by the short inter­actions described above.

**Figure 6:**
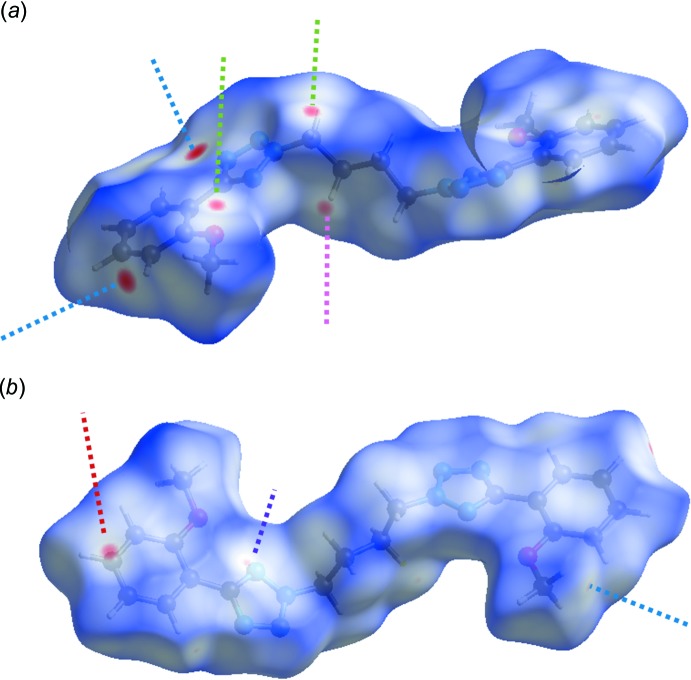
*d*_norm_ mapped on the Hirshfeld surface for visualizing the inter­molecular inter­actions.(*a*) front side, (*b*) back side.

To provide an overall view of the weak inter­actions between the mol­ecules, a Hirshfeld surface analysis (Spackman & Jayatilaka, 2009 ▸) was performed with *CrystalExplorer17* (Turner *et al.*, 2017 ▸). The Hirshfeld surface was calculated using a standard (high) surface resolution with the three-dimensional (3D) *d*_norm_ surface plotted over a fixed colour scale of −0.1339 (red) to 1.4773 a.u. (blue). The 3D *d*_norm_ surface of the title complex is shown in Fig. 6 ▸ *a* and 6*b*. The red spots indicate short contacts, *i.e.*, negative *d*_norm_ values on the surface, which highlight the most important weak inter­actions: C17—H17*A*⋯O1^i^ hydrogen bond (green dashed line), C4—H4⋯C1^iv^ contact (blue in Fig. 6 ▸ *a*), C18—H18*B*⋯C5^iii^ (pink in Fig. 6 ▸ *a*, red in Fig. 6 ▸ *b*) and C16—H16*A*⋯C1^v^ (blue in Fig. 6 ▸ *b*).

## Database survey   

A search of the Cambridge Structural Database (CSD Version 5.40, November 2018; Groom *et al.*, 2016 ▸) for bis­(tetra­zol­yl)alkane fragments provided four hits with a methyl­ene bridge [SAVPAJ, SAVPIR (Freis *et al.*, 2017 ▸), OYIWOK02 (Feng, Qiu *et al.*, 2016 ▸) and UMOJEN (Feng, Bi *et al.*, 2016 ▸)] and two with a propyl­ene bridge (SIBFIV, SIBFUH; Wurzenberger *et al.*, 2018 ▸). The butyl­ene-bridged examples include a bis­tetra­zolyl copper complex (SIBGIW; Wurzenberger *et al.*, 2018 ▸) and three bis­(pyridyl­tetra­zol­yl)silver complexes (QOKBAV, QOKBEZ, QOKBID; Wang *et al.*, 2014 ▸). All of the above bis­(tetra­zol­yl)alkane structures are metal complexes. It is worth noting that inter­esting metal-free cyclic bis­tetra­zolyl compounds have been reported (VELPUZ, VELPOT; Voitekhovich *et al.*, 2012 ▸) in which the bis­(tetra­zol­yl)butane fragment is part of a ring.

## Synthesis and crystallization   

The synthesis scheme for the title compound is represented in Fig. 1 ▸. The sodium salt of 5-(2-meth­oxy­phen­yl)-1*H*-tetra­zole (495 mg, 2.5 mmol) and di­bromo­butane (150 µl, 1.25 mmol) were dissolved in aceto­nitrile and refluxed for 2 d. The resulting white solid was filtered and the solvent was removed under reduced pressure. The residue was purified by column chromatography on silica gel using hexa­ne:acetone (1:1) as eluent. Three isomeric compounds were obtained, as shown in Fig. 1 ▸. The major product (I) (yield = 35%) was recrystallized in ethanol by the slow evaporation method and yielded colourless crystals of the title compound.

Spectroscopic data: ^1^H NMR (DMSO, 400 MHz): δ = 7.62 (*t*, 2H, Ph), 7.36 (*d*, 2H, Ph), 7.22 (*d*, 2H, Ph), 7.12 (*t*, 2H, Ph), 4.13 (*s*, 4H, CH_2_), 3.71 (*s*, 6H, OCH_3_), 1.66 (*s*, 4H, CH_2_). ^13^C NMR (125 MHz, DMSO): 156.56, 152.18, 133.10, 131.20, 120.80, 112.26, 111.91, 55.50, 46.63, 25.57 ppm.

## Refinement   

Crystal data, data collection and structure refinement details are summarized in Table 2 ▸. All H atoms were included in calculated positions using a riding model, with C—H = 0.95–1.00 Å and *U*_iso_(H) = 1.5*U*_eq_(C) for methyl H atoms and *U*_iso_(H) = 1.2*U*_eq_(C) for all others. Two reflections (100 and 110) were omitted because of truncation by the beamstop.

## Supplementary Material

Crystal structure: contains datablock(s) I. DOI: 10.1107/S2056989019014877/fy2138sup1.cif

Structure factors: contains datablock(s) I. DOI: 10.1107/S2056989019014877/fy2138Isup2.hkl

Supporting information file. DOI: 10.1107/S2056989019014877/fy2138Isup3.cdx

Supporting information file. DOI: 10.1107/S2056989019014877/fy2138Isup4.cml

CCDC references: 1963337, 1963337

Additional supporting information:  crystallographic information; 3D view; checkCIF report

## Figures and Tables

**Table 1:** Hydrogen-bond geometry (Å, °)

*D*—H⋯*A*	*D*—H	H⋯*A*	*D*⋯*A*	*D*—H⋯*A*
C7—H7⋯N4	0.95	2.48	2.8371 (16)	102
C15—H15⋯N8	0.95	2.53	2.8586 (17)	101
C17—H17*A*⋯O1^i^	0.99	2.58	3.4337 (15)	144

Symmetry code: (i) 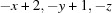.

**Table 2:** Experimental details

Crystal data	
Chemical formula	C_20_H_22_N_8_O_2_
*M*_r_	406.45
Crystal system, space group	Monoclinic, *P*2_1_/*c*
Temperature (K)	100
*a*, *b*, *c* (Å)	13.2904 (2), 10.2785 (2), 14.4968 (3)
β (°)	100.2538 (9)
*V* (Å^3^)	1948.71 (6)
*Z*	4
Radiation type	Mo *K*α
μ (mm^−1^)	0.10
Crystal size (mm)	0.1 × 0.1 × 0.08

Data collection	
Diffractometer	Bruker APEXII CCD
Absorption correction	Multi-scan (*SADABS*; Krause *et al.*, 2015 ▸)
*T*_min_, *T*_max_	0.706, 0.745
No. of measured, independent and observed [*I* > 2σ(*I*)] reflections	26494, 4008, 3516
*R*_int_	0.021
(sin θ/λ)_max_ (Å^−1^)	0.627

Refinement	
*R*[*F* ^2^ > 2σ(*F* ^2^)], *wR*(*F* ^2^), *S*	0.037, 0.098, 1.05
No. of reflections	4008
No. of parameters	273
H-atom treatment	H-atom parameters constrained
Δρ_max_, Δρ_min_ (e Å^−3^)	0.25, −0.37

Computer programs: *APEX2* and *SAINT* (Bruker, 2014 ▸), *SHELXS* (Sheldrick, 2008 ▸), *SHELXL* (Sheldrick, 2015 ▸), *Mercury* (Macrae *et al.*, 2008 ▸) and *OLEX2* (Dolomanov *et al.*, 2009 ▸).

## supplementary crystallographic information

### Crystal data

**Table:**

C_20_H_22_N_8_O_2_	*F*(000) = 856
*M**_r_* = 406.45	*D*_x_ = 1.385 Mg m^−^^3^
Monoclinic, *P*2_1_/*c*	Mo *K*α radiation, λ = 0.71073 Å
*a* = 13.2904 (2) Å	Cell parameters from 9933 reflections
*b* = 10.2785 (2) Å	θ = 2.5–26.4°
*c* = 14.4968 (3) Å	µ = 0.10 mm^−^^1^
β = 100.2538 (9)°	*T* = 100 K
*V* = 1948.71 (6) Å^3^	Block, colorless
*Z* = 4	0.1 × 0.1 × 0.08 mm

### Data collection

**Table:**

Bruker APEXII CCD diffractometer	3516 reflections with *I* > 2σ(*I*)
φ and ω scans	*R*_int_ = 0.021
Absorption correction: multi-scan (SADABS; Krause *et al.*, 2015)	θ_max_ = 26.5°, θ_min_ = 2.4°
*T*_min_ = 0.706, *T*_max_ = 0.745	*h* = −16→16
26494 measured reflections	*k* = −12→12
4008 independent reflections	*l* = −18→18

### Refinement

**Table:**

Refinement on *F*^2^	Primary atom site location: structure-invariant direct methods
Least-squares matrix: full	Secondary atom site location: difference Fourier map
*R*[*F*^2^ > 2σ(*F*^2^)] = 0.037	Hydrogen site location: inferred from neighbouring sites
*wR*(*F*^2^) = 0.098	H-atom parameters constrained
*S* = 1.05	*w* = 1/[σ^2^(*F*_o_^2^) + (0.0491*P*)^2^ + 0.7236*P*] where *P* = (*F*_o_^2^ + 2*F*_c_^2^)/3
4008 reflections	(Δ/σ)_max_ = 0.001
273 parameters	Δρ_max_ = 0.25 e Å^−^^3^
0 restraints	Δρ_min_ = −0.37 e Å^−^^3^

### Special details

**Table:**

Geometry. All esds (except the esd in the dihedral angle between two l.s. planes) are estimated using the full covariance matrix. The cell esds are taken into account individually in the estimation of esds in distances, angles and torsion angles; correlations between esds in cell parameters are only used when they are defined by crystal symmetry. An approximate (isotropic) treatment of cell esds is used for estimating esds involving l.s. planes.
Refinement. 1. Fixed Uiso At 1.2 times of: All C(H) groups, All C(H,H) groups At 1.5 times of: All C(H,H,H) groups 2.a Secondary CH2 refined with riding coordinates: C18(H18A,H18B), C19(H19A,H19B), C17(H17A,H17B), C20(H20A,H20B) 2.b Aromatic/amide H refined with riding coordinates: C7(H7), C13(H13), C15(H15), C4(H4), C6(H6), C12(H12), C5(H5), C14(H14) 2.c Idealised Me refined as rotating group: C8(H8A,H8B,H8C), C16(H16A,H16B,H16C)

### Fractional atomic coordinates and isotropic or equivalent isotropic displacement parameters (Å^2^)

**Table:**

	*x*	*y*	*z*	*U*_iso_*/*U*_eq_	
O1	0.95431 (7)	0.24652 (8)	0.11811 (6)	0.0258 (2)	
N1	0.86468 (7)	0.47789 (10)	0.06134 (7)	0.0217 (2)	
N2	0.81869 (8)	0.58734 (10)	0.02753 (7)	0.0217 (2)	
N5	0.60298 (7)	0.58496 (10)	−0.44500 (7)	0.0226 (2)	
N6	0.68182 (8)	0.51604 (10)	−0.40215 (7)	0.0247 (2)	
N4	0.77518 (8)	0.57102 (11)	0.15994 (7)	0.0269 (2)	
C3	0.92713 (9)	0.26068 (12)	0.20399 (8)	0.0220 (3)	
C1	0.83621 (8)	0.46963 (11)	0.14486 (8)	0.0198 (2)	
N3	0.76475 (8)	0.64445 (11)	0.08397 (7)	0.0278 (2)	
C9	0.62616 (9)	0.59998 (12)	−0.52995 (8)	0.0220 (2)	
O2	0.46627 (8)	0.77149 (12)	−0.51135 (6)	0.0463 (3)	
C10	0.56607 (9)	0.67077 (12)	−0.60922 (8)	0.0215 (2)	
N8	0.71619 (9)	0.54247 (13)	−0.53723 (8)	0.0348 (3)	
C7	0.83412 (9)	0.38359 (12)	0.30302 (8)	0.0238 (3)	
H7	0.7911	0.4547	0.3117	0.029*	
C2	0.86647 (9)	0.36911 (12)	0.21675 (8)	0.0207 (2)	
C13	0.46290 (10)	0.80750 (13)	−0.76430 (9)	0.0284 (3)	
H13	0.4280	0.8544	−0.8169	0.034*	
C18	0.74738 (9)	0.55296 (12)	−0.13870 (8)	0.0232 (3)	
H18A	0.6763	0.5728	−0.1314	0.028*	
H18B	0.7596	0.4593	−0.1251	0.028*	
C19	0.75877 (9)	0.57977 (12)	−0.23988 (8)	0.0242 (3)	
H19A	0.7355	0.6692	−0.2580	0.029*	
H19B	0.8314	0.5718	−0.2464	0.029*	
C15	0.59024 (9)	0.65380 (12)	−0.69829 (8)	0.0237 (3)	
H15	0.6431	0.5947	−0.7061	0.028*	
C4	0.95664 (9)	0.17429 (12)	0.27783 (9)	0.0268 (3)	
H4	0.9983	0.1016	0.2696	0.032*	
C6	0.86318 (10)	0.29699 (13)	0.37594 (9)	0.0282 (3)	
H6	0.8405	0.3085	0.4339	0.034*	
N7	0.75068 (9)	0.48940 (13)	−0.45441 (8)	0.0366 (3)	
C17	0.82070 (9)	0.63252 (12)	−0.06795 (8)	0.0240 (3)	
H17A	0.8909	0.6243	−0.0814	0.029*	
H17B	0.8010	0.7255	−0.0736	0.029*	
C12	0.43688 (10)	0.82663 (13)	−0.67683 (9)	0.0299 (3)	
H12	0.3844	0.8866	−0.6698	0.036*	
C11	0.48755 (10)	0.75822 (13)	−0.59921 (8)	0.0276 (3)	
C8	1.00848 (10)	0.13020 (13)	0.10279 (10)	0.0302 (3)	
H8A	1.0215	0.1294	0.0383	0.045*	
H8B	0.9673	0.0541	0.1129	0.045*	
H8C	1.0737	0.1275	0.1467	0.045*	
C5	0.92583 (10)	0.19321 (13)	0.36327 (9)	0.0289 (3)	
H5	0.9478	0.1346	0.4135	0.035*	
C20	0.69416 (10)	0.48127 (13)	−0.30300 (8)	0.0259 (3)	
H20A	0.7268	0.3946	−0.2934	0.031*	
H20B	0.6259	0.4753	−0.2849	0.031*	
C14	0.53931 (10)	0.72068 (13)	−0.77559 (8)	0.0265 (3)	
H14	0.5566	0.7071	−0.8357	0.032*	
C16	0.38052 (17)	0.8497 (2)	−0.50095 (12)	0.0780 (8)	
H16A	0.3195	0.8174	−0.5428	0.117*	
H16B	0.3934	0.9400	−0.5170	0.117*	
H16C	0.3697	0.8453	−0.4359	0.117*	

### Atomic displacement parameters (Å^2^)

**Table:**

	*U*^11^	*U*^22^	*U*^33^	*U*^12^	*U*^13^	*U*^23^
O1	0.0290 (5)	0.0251 (4)	0.0246 (4)	0.0063 (4)	0.0083 (4)	0.0012 (3)
N1	0.0245 (5)	0.0214 (5)	0.0190 (5)	0.0009 (4)	0.0038 (4)	0.0011 (4)
N2	0.0242 (5)	0.0221 (5)	0.0186 (5)	0.0009 (4)	0.0035 (4)	0.0003 (4)
N5	0.0233 (5)	0.0256 (5)	0.0184 (5)	0.0025 (4)	0.0024 (4)	0.0011 (4)
N6	0.0260 (5)	0.0293 (6)	0.0184 (5)	0.0058 (4)	0.0030 (4)	0.0014 (4)
N4	0.0316 (6)	0.0290 (6)	0.0208 (5)	0.0073 (4)	0.0070 (4)	0.0029 (4)
C3	0.0189 (5)	0.0251 (6)	0.0217 (6)	−0.0028 (4)	0.0029 (4)	0.0003 (5)
C1	0.0183 (5)	0.0222 (6)	0.0186 (5)	−0.0020 (4)	0.0024 (4)	−0.0023 (4)
N3	0.0332 (6)	0.0288 (6)	0.0227 (5)	0.0071 (5)	0.0082 (4)	0.0018 (4)
C9	0.0234 (6)	0.0241 (6)	0.0185 (6)	0.0016 (5)	0.0037 (4)	−0.0030 (5)
O2	0.0503 (6)	0.0703 (8)	0.0190 (5)	0.0395 (6)	0.0080 (4)	0.0032 (5)
C10	0.0227 (6)	0.0234 (6)	0.0181 (6)	−0.0002 (5)	0.0024 (4)	−0.0010 (5)
N8	0.0351 (6)	0.0489 (7)	0.0212 (5)	0.0174 (5)	0.0067 (5)	0.0042 (5)
C7	0.0229 (6)	0.0275 (6)	0.0208 (6)	−0.0020 (5)	0.0032 (5)	−0.0014 (5)
C2	0.0192 (5)	0.0233 (6)	0.0188 (6)	−0.0028 (4)	0.0015 (4)	0.0004 (5)
C13	0.0335 (7)	0.0285 (6)	0.0214 (6)	−0.0001 (5)	0.0000 (5)	0.0046 (5)
C18	0.0253 (6)	0.0254 (6)	0.0188 (6)	0.0001 (5)	0.0040 (5)	0.0036 (5)
C19	0.0268 (6)	0.0267 (6)	0.0193 (6)	0.0018 (5)	0.0047 (5)	0.0038 (5)
C15	0.0265 (6)	0.0242 (6)	0.0213 (6)	−0.0004 (5)	0.0067 (5)	−0.0012 (5)
C4	0.0221 (6)	0.0264 (6)	0.0313 (7)	0.0007 (5)	0.0030 (5)	0.0052 (5)
C6	0.0283 (6)	0.0363 (7)	0.0200 (6)	−0.0044 (5)	0.0047 (5)	0.0025 (5)
N7	0.0370 (6)	0.0523 (8)	0.0214 (5)	0.0202 (6)	0.0075 (5)	0.0051 (5)
C17	0.0292 (6)	0.0245 (6)	0.0189 (6)	−0.0004 (5)	0.0062 (5)	0.0041 (5)
C12	0.0311 (7)	0.0320 (7)	0.0254 (6)	0.0100 (5)	0.0019 (5)	0.0018 (5)
C11	0.0293 (6)	0.0345 (7)	0.0187 (6)	0.0067 (5)	0.0035 (5)	−0.0008 (5)
C8	0.0318 (7)	0.0250 (6)	0.0356 (7)	0.0057 (5)	0.0107 (6)	−0.0010 (5)
C5	0.0259 (6)	0.0338 (7)	0.0256 (6)	−0.0028 (5)	0.0008 (5)	0.0098 (5)
C20	0.0304 (6)	0.0292 (7)	0.0174 (6)	0.0015 (5)	0.0026 (5)	0.0046 (5)
C14	0.0335 (7)	0.0288 (6)	0.0177 (6)	−0.0033 (5)	0.0060 (5)	0.0005 (5)
C16	0.0869 (14)	0.1223 (19)	0.0290 (8)	0.0798 (14)	0.0218 (9)	0.0134 (10)

### Geometric parameters (Å, º)

**Table:**

O1—C3	1.3644 (14)	C18—H18A	0.9900
O1—C8	1.4331 (15)	C18—H18B	0.9900
N1—N2	1.3315 (14)	C18—C19	1.5262 (16)
N1—C1	1.3339 (15)	C18—C17	1.5214 (17)
N2—N3	1.3179 (14)	C19—H19A	0.9900
N2—C17	1.4647 (14)	C19—H19B	0.9900
N5—N6	1.3243 (14)	C19—C20	1.5232 (17)
N5—C9	1.3306 (15)	C15—H15	0.9500
N6—N7	1.3166 (15)	C15—C14	1.3843 (17)
N6—C20	1.4618 (15)	C4—H4	0.9500
N4—C1	1.3619 (15)	C4—C5	1.3858 (18)
N4—N3	1.3218 (15)	C6—H6	0.9500
C3—C2	1.4069 (17)	C6—C5	1.3854 (19)
C3—C4	1.3924 (17)	C17—H17A	0.9900
C1—C2	1.4719 (16)	C17—H17B	0.9900
C9—C10	1.4700 (16)	C12—H12	0.9500
C9—N8	1.3555 (16)	C12—C11	1.3945 (18)
O2—C11	1.3597 (15)	C8—H8A	0.9800
O2—C16	1.4242 (18)	C8—H8B	0.9800
C10—C15	1.3959 (16)	C8—H8C	0.9800
C10—C11	1.4043 (17)	C5—H5	0.9500
N8—N7	1.3240 (16)	C20—H20A	0.9900
C7—H7	0.9500	C20—H20B	0.9900
C7—C2	1.4009 (16)	C14—H14	0.9500
C7—C6	1.3833 (18)	C16—H16A	0.9800
C13—H13	0.9500	C16—H16B	0.9800
C13—C12	1.3864 (18)	C16—H16C	0.9800
C13—C14	1.3834 (18)		
			
C3—O1—C8	116.89 (10)	C10—C15—H15	119.1
N2—N1—C1	101.66 (9)	C14—C15—C10	121.79 (11)
N1—N2—C17	122.10 (10)	C14—C15—H15	119.1
N3—N2—N1	114.36 (9)	C3—C4—H4	119.7
N3—N2—C17	123.30 (10)	C5—C4—C3	120.67 (12)
N6—N5—C9	101.65 (9)	C5—C4—H4	119.7
N5—N6—C20	122.20 (10)	C7—C6—H6	120.4
N7—N6—N5	114.48 (10)	C7—C6—C5	119.12 (11)
N7—N6—C20	123.17 (10)	C5—C6—H6	120.4
N3—N4—C1	106.28 (10)	N6—N7—N8	105.79 (10)
O1—C3—C2	117.10 (10)	N2—C17—C18	110.45 (9)
O1—C3—C4	123.32 (11)	N2—C17—H17A	109.6
C4—C3—C2	119.58 (11)	N2—C17—H17B	109.6
N1—C1—N4	111.73 (10)	C18—C17—H17A	109.6
N1—C1—C2	126.98 (10)	C18—C17—H17B	109.6
N4—C1—C2	121.26 (10)	H17A—C17—H17B	108.1
N2—N3—N4	105.96 (10)	C13—C12—H12	119.9
N5—C9—C10	126.72 (10)	C13—C12—C11	120.22 (12)
N5—C9—N8	111.99 (10)	C11—C12—H12	119.9
N8—C9—C10	121.28 (10)	O2—C11—C10	116.32 (11)
C11—O2—C16	117.36 (11)	O2—C11—C12	123.66 (11)
C15—C10—C9	118.61 (11)	C12—C11—C10	120.02 (11)
C15—C10—C11	118.25 (11)	O1—C8—H8A	109.5
C11—C10—C9	123.11 (10)	O1—C8—H8B	109.5
N7—N8—C9	106.08 (10)	O1—C8—H8C	109.5
C2—C7—H7	119.1	H8A—C8—H8B	109.5
C6—C7—H7	119.1	H8A—C8—H8C	109.5
C6—C7—C2	121.70 (12)	H8B—C8—H8C	109.5
C3—C2—C1	123.62 (10)	C4—C5—H5	119.8
C7—C2—C3	118.41 (11)	C6—C5—C4	120.45 (12)
C7—C2—C1	117.97 (11)	C6—C5—H5	119.8
C12—C13—H13	119.7	N6—C20—C19	112.38 (10)
C14—C13—H13	119.7	N6—C20—H20A	109.1
C14—C13—C12	120.50 (12)	N6—C20—H20B	109.1
H18A—C18—H18B	107.8	C19—C20—H20A	109.1
C19—C18—H18A	109.0	C19—C20—H20B	109.1
C19—C18—H18B	109.0	H20A—C20—H20B	107.9
C17—C18—H18A	109.0	C13—C14—C15	119.21 (11)
C17—C18—H18B	109.0	C13—C14—H14	120.4
C17—C18—C19	112.93 (10)	C15—C14—H14	120.4
C18—C19—H19A	110.0	O2—C16—H16A	109.5
C18—C19—H19B	110.0	O2—C16—H16B	109.5
H19A—C19—H19B	108.4	O2—C16—H16C	109.5
C20—C19—C18	108.39 (10)	H16A—C16—H16B	109.5
C20—C19—H19A	110.0	H16A—C16—H16C	109.5
C20—C19—H19B	110.0	H16B—C16—H16C	109.5

### Hydrogen-bond geometry (Å, º)

**Table:**

*D*—H···*A*	*D*—H	H···*A*	*D*···*A*	*D*—H···*A*
C7—H7···N4	0.95	2.48	2.8371 (16)	102
C15—H15···N8	0.95	2.53	2.8586 (17)	101
C17—H17*A*···O1^i^	0.99	2.58	3.4337 (15)	144

Symmetry code: (i) −*x*+2, −*y*+1, −*z*.

## References

[bb1] Boland, Y., Safin, D. A., Tinant, B., Babashkina, M. G., Marchand-Brynaert, J. & Garcia, Y. (2013). *New J. Chem.* **37**, 1174–1179.

[bb2] Bruker (2014). *APEX2* and *SAINT*. Bruker AXS Inc., Madison, Wisconsin, USA.

[bb3] Dolomanov, O. V., Bourhis, L. J., Gildea, R. J., Howard, J. A. K. & Puschmann, H. (2009). *J. Appl. Cryst.* **42**, 339–341.

[bb4] Fan, J.-Z., Du, C.-C. & Wang, D.-Z. (2016). *Polyhedron*, **117**, 487–495.

[bb5] Feng, Y.-A., Qiu, H., Yang, S.-A., Du, J. & Zhang, T.-L. (2016). *Dalton Trans.* **45**, 17117–17122. 10.1039/c6dt03271k 27766333

[bb6] Feng, Y., Bi, Y., Zhao, W. & Zhang, T. (2016). *J. Mater. Chem. A*, **4**, 7596–7600.

[bb7] Freis, M., Klapötke, T. M., Stierstorfer, J. & Szimhardt, N. (2017). *Inorg. Chem.* **56**, 7936–7947. 10.1021/acs.inorgchem.7b00432 28653835

[bb8] Groom, C. R., Bruno, I. J., Lightfoot, M. P. & Ward, S. C. (2016). *Acta Cryst.* B**72**, 171–179. 10.1107/S2052520616003954 PMC4822653 27048719

[bb9] Karaghiosoff, K., Klapötke, T. M. & Miró Sabaté, C. (2009). *Chem. Eur. J.* **15**, 1164–1176. 10.1002/chem.200801666 19105192

[bb10] Krause, L., Herbst-Irmer, R., Sheldrick, G. M. & Stalke, D. (2015). *J. Appl. Cryst.* **48**, 3–10. 10.1107/S1600576714022985 PMC4453166 26089746

[bb11] Lee, S. G., Ryu, J. Y. & Lee, J. (2017). *Acta Cryst.* E**73**, 1971–1973. 10.1107/S205698901701698X PMC5730263 29250426

[bb12] Liu, Z.-Y., Zou, H.-A., Hou, Z.-J., Yang, E.-C. & Zhao, X.-J. (2013). *Dalton Trans.* **42**, 15716–15725. 10.1039/c3dt52190g 24048211

[bb13] Macrae, C. F., Bruno, I. J., Chisholm, J. A., Edgington, P. R., McCabe, P., Pidcock, E., Rodriguez-Monge, L., Taylor, R., van de Streek, J. & Wood, P. A. (2008). *J. Appl. Cryst.* **41**, 466–470.

[bb14] Sheldrick, G. M. (2008). *Acta Cryst.* A**64**, 112–122. 10.1107/S0108767307043930 18156677

[bb15] Sheldrick, G. M. (2015). *Acta Cryst.* C**71**, 3–8.

[bb16] Spackman, M. A. & Jayatilaka, D. (2009). *CrystEngComm*, **11**, 19–32.

[bb17] Tăbăcaru, A., Pettinari, C. & Galli, S. (2018). *Coord. Chem. Rev.* **372**, 1–30.

[bb18] Turner, M. J., McKinnon, J. J., Wolff, S. K., Grimwood, D. J., Spackman, P. R., Jayatilaka, D. & Spackman, M. A. (2017). *CrystalExplorer17*. University of Western Australia. http://hirshfeldsurface.net.

[bb19] Voitekhovich, S. V., Lyakhov, A. S., Ivashkevich, L. S. & Gaponik, P. N. (2012). *Tetrahedron Lett.* **53**, 6111–6114.

[bb20] Wang, X.-L., Li, N., Tian, A.-X., Ying, J., Li, T.-J., Lin, X.-L., Luan, J. & Yang, Y. (2014). *Inorg. Chem.* **53**, 7118–7129. 10.1021/ic403153f 24985955

[bb21] Wurzenberger, M. H. H., Szimhardt, N. & Stierstorfer, J. (2018). *J. Am. Chem. Soc.* **140**, 3206–3209. 10.1021/jacs.7b13230 29451790

[bb22] Zhao, H., Qu, Z.-R., Ye, H.-Y. & Xiong, R.-G. (2008). *Chem. Soc. Rev.* **37**, 84–100. 10.1039/b616738c 18197335

[bb23] Zhao, Y.-P., Li, Y., Cui, C.-Y., Xiao, Y., Li, R., Wang, S.-H., Zheng, F.-K. & Guo, G.-C. (2016). *Inorg. Chem.* **55**, 7335–7340. 10.1021/acs.inorgchem.6b00320 27400274

